# Creating expert patients: outcomes from a national digital therapeutic approach for people with asthma in Wales

**DOI:** 10.1038/s41533-025-00433-x

**Published:** 2025-06-09

**Authors:** Simon M. Barry, Julian Forton, Gareth R. Davies, Gwyneth A. Davies, Katie Pink, Alison Whittaker, Jerome Donagh, Dan Menzies, Mark Andrews, Grace Moore, Chris Davies

**Affiliations:** 1 Respiratory Medicine, Cardiff and Vale Health Board, Cardiff, UK; 2 School of Medicine, Cardiff University, Cardiff, UK; 3 Children’s Hospital for Wales, Cardiff, UK; 4 Institute of Clinical Science and Technology, Cardiff, UK; 5 Population Data Science, Faculty of Medicine, Health and Life Science, Swansea University, Swansea, UK; 6 General practice, Bridgend, UK; 7 Respiratory Medicine, Betsi Cadwalladr Health Board, Rhyl, UK; 8 Respiratory Medicine, Hywel Dda Health Board, Llanelli, UK

**Keywords:** Diseases, Health care

## Abstract

National applications (apps) for adults with asthma were implemented as part of a respiratory toolkit across Wales from 2020. Data were collected on patient recorded asthma control including the Royal College of Physicians three questions. All general practices in Wales had patients registered on the asthma app and by September 2024, 12,567 (57.8%) of patients who downloaded the app went on to register. Analysis comparing baseline with four or more months of app use demonstrated improvements in the percent of those having a Royal College of Physicians asthma score of 0 (26.5% vs 40.7%, *p* = 0.0011), together with improvements in those not using a reliever inhaler at all (29.1% vs 39.2%, *p* = 0.0001). Where we had paired data one year apart, the improvements in asthma control were greater in those from most deprived areas. For those who used the app there were improvements across important metrics of asthma control consistent with better patient self-management.

## Introduction

The 2017/8 Royal College of Physicians (RCP) report of primary care outcomes for patients with asthma and chronic obstructive pulmonary disease (COPD) in Wales demonstrated significant failure to deliver basics aspects of care^1^. For example, asthma self-management plans which have been identified as crucial for improving asthma outcomes^2–5^ and recommended as a tool for reducing asthma deaths^6^ were only being used by 25% of people with asthma in Wales. In addition, only 49% of patients attending annual asthma reviews had their inhaler technique checked. A follow up audit on 2020 data in Wales^7^ showed no improvements in these outcomes and also recorded a significant over usage of short acting B2 agonists, another factor associated with adverse asthma outcomes^8^.

These findings suggested to us that a different approach was required to change outcomes for people with asthma. As a result, we created a national respiratory toolkit for Wales comprising patient facing apps for adults and children with asthma as well as patients with COPD, national asthma guidelines, educational modules for healthcare professionals to standardise annual asthma reviews, and a suite of quality improvement tools. Together, this approach falls under the rubric of digital therapeutics which is defined as ‘health softwares intended to treat or alleviate a disease, disorder, condition, or injury by generating and delivering a medical intervention that has a demonstrable positive therapeutic impact on a patient’s health’^9^.

Implementation of the toolkit was promoted from September 2022 across the whole of primary and secondary care in Wales using an established digital implementation framework that had been previously successfully used during the COVID pandemic^10^ and for screening Ukrainian refugees for TB^11^. The underlying principle of this approach was to empower patients with the knowledge and skills to better manage their asthma.

Here we present results from an initial evaluation of this national intervention in adults, focussing on patient reported outcome measures (PROMS) of asthma control.

## Methods

### Apps

The Asthmahub app for adults is a digital therapeutic app which was developed by the Institute for Clinical Science and Technology (ICST) in collaboration with senior clinicians in Wales. The app has been developed, tested and refined in collaboration with patient testers, to optimise usability. Close alignment with patient groups such as Asthma and Lung UK and Breath Easy has further improved the apps co-production. Feedback is continuously gathered through regular surveys as well as a dedicated support line, allowing users to share their experiences and suggestions for improvement. The asthma app is a Class I Medical Device registered with the medicines and healthcare products agency (MHRA, product reference 9213), free for patients in Wales, available in both Welsh and English and was launched in July 2020. Key features include a self-management algorithm, instructional videos on proper inhaler use, and educational videos on multiple aspects of asthma care. Users are invited to complete a monthly 10-question asthma checker to assess aspects of their asthma control, with reminders sent to prompt them (Table 1).

**Table 1:** Monthly asthma checker questions for asthma app users.

Question number	Question
1	Did you have an asthma annual review this month?
2	Did you pick up your asthma prescription?
3	How many times did you visit your GP for your Asthma?
4	How many times did you go to A&E for your Asthma?
5	How many times were you admitted to hospital for your Asthma?
6	Did you have a hospital out-patient appointment last month?
7	How many courses of prednisolone did you have?
8	On average, how many times are you using your reliever inhaler per week?
9	Does anyone in your household smoke?
10: RCP 1	Have you had difficulty sleeping because of your asthma symptoms?
10: RCP 2	Have you had your usual asthma symptoms during the day?
10: RCP 3	Has your asthma interfered with your usual activities?

### Implementation

The apps were disseminated utilising a Simple Implementation Science (SIMPSI) framework created by ICST^12^. This has been described elsewhere^13^, but briefly involved a formal structured implementation process at national level utilising phases for development (creating the apps), instillation (installing the apps into a test environment), scaling (maximising uptake of the apps) and sustainability (ensuring widespread adoption and acceptance). This process is ongoing in Wales.

### Data collection

We analysed data on people with asthma using the adult asthma app. Upon initial sign up to the apps, patients agree that their anonymised data can be utilised for research purposes, to better understand the population with asthma in Wales.

Available data includes age and smoking status, location of the patients’ primary care practice and types of inhalers used. App users are asked to complete a monthly asthma checker to assess self-reported symptoms and control which include the Royal College of Physicians three questions^14^ (RCP3Q). Sex of the users was not recorded.

Users were grouped by relative deprivation at GP practice level, based on the percentage of patients registered to their GP practice living within the most deprived quintile of areas in Wales, according to the Welsh Index of Multiple Deprivation (WIMD)^15^. The practices were divided into the 30% of practices with the highest percentages in the most deprived quintile, the 20% of practices with next highest percentages in the most deprived quintile, and the 50% of practices with the lowest percentages in the most deprived quintile, an aggregation in line with the recommended groupings for WIMD.

Two subgroups were analysed. For the first subgroup, all app users with one or more app use four or more months after their first app use were identified. For this subgroup, their baseline scores were compared en masse with all their four or more months later scores. For the second subgroup, app users who had recorded an RCP score both at baseline and exactly 12 months later were identified for paired analysis.

### Statistical analysis

For app users using the app for at least four months, comparisons of their RCP3Q scores and reliever inhaler use at first app use and at app uses four or more months later were made using the two sample test of proportions in Stata (prtest). Smoking proportions were compared between deprivation groups using the Chi Square test. The change in RCP score at 12 months for paired observations was evaluated using the paired t-test. All analyses were conducted in Stata 18.

## Results

### Asthma app uptake

**Fig. 1:**
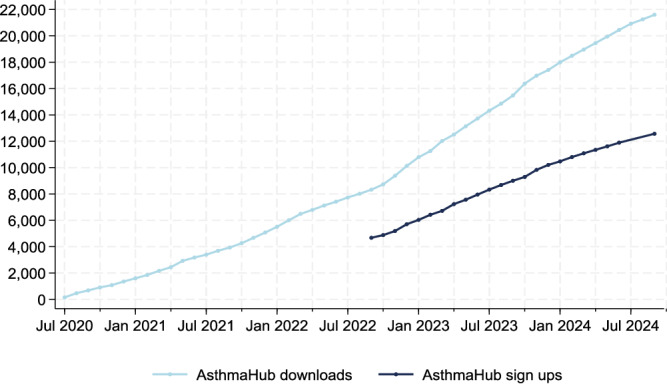
Cumulative asthma hub downloads and signatories from July 2020 until September 2024.

There was a continued increase in asthma app downloads over time with an increase in the download rate from October 2022 (Fig. 1). The number of registrations was collected from September 2022. By September 2024, 21,745 people with asthma living in Wales had downloaded the app and 12,567 (57.8%) completed registration. The mean (standard deviation) of monthly downloads up until October 2022 were 311 (92), and after this time there was a 75% increase to 545 (147). This increase is as a result of a change in GP prescribing behaviour. GP practices requested that their patients with asthma download the app either during in-person consultations, or via mass communications such as text messaging or email.

App users were registered with 378 different practices in Wales. Branch surgery data was amalgamated with that from the main surgery. As of October 29th 2024 there were 373 registered main practices in Wales^16^ suggesting that we had data from all practices. The median number of asthma app signatories per GP practice was 21, IQR 10–46, with notable outlier practices having up to 246.

### Demographics and asthma app use

Demographic data recorded a roughly even distribution of app registrations for the age deciles from 18–69 with an expected reduction in those over 70 (Table 2). 1432 (25.5%) of app users came from the most deprived 30% of practices. Overall, 17.3% of app users described themselves as current smokers, slightly above the national prevalence in Wales of 16%. Smoking proportions were higher in the most deprived 30% of practices (22.3%) compared to the next most deprived 20% (19.2%) and also to the least deprived 50% (14.2%, Chi square 47.1, *p* < 0.0001). Of those who signed up to the app, nearly half (47.7%) never used it. For the remaining 5657 who used the app, 24.5% used it once, 65.6% used it 2–6 times, 6.7% 7–12 times and 3.1% more than 13 times.

**Table 2:** Demographic data, smoking status and app usage of the study population.

Characteristic	Level	App uses										
		0		1		2–6		7–12		13+		Total
		n	*%*	n	*%*	n	*%*	n	*%*	n	*%*	
Age	18–29	959	49.0	275	14.1	673	34.4	38	1.9	12	0.6	1957
	30–39	1163	51.3	282	12.4	747	32.9	61	2.7	15	0.7	2268
	40–49	970	45.1	274	12.8	803	37.4	65	3.0	37	1.7	2149
	50–59	1048	46.4	274	12.1	811	35.9	96	4.3	32	1.4	2261
	60–69	681	42.5	192	12.0	595	37.1	87	5.4	49	3.1	1604
	70+	347	44.1	91	11.6	283	36.0	33	4.2	33	4.2	787
	missing	20	55.6	3	8.3	11	30.6	2	5.6	0	0.0	36
	All	5188	46.9	1391	12.6	3923	35.5	382	3.5	178	1.6	11,062
Practice deprivation group	Most 30%	1346	48.5	327	11.8	993	35.8	77	2.8	35	1.3	2778
	Next 20%	955	48.1	261	13.2	671	33.8	65	3.3	32	1.6	1984
	Least 50%	2731	45.7	757	12.7	2151	36.0	233	3.9	110	1.8	5982
	missing	156	49.1	46	14.5	107	34.0	7	2.2	1	0.3	318
	All	5188	46.9	1391	12.6	3923	35.5	382	3.5	178	1.6	11,062
Smoking status	Not smoker	0	0.0	906	19.9	3175	69.9	326	7.2	155	3.4	4562
	Smoker	0	0.0	226	23.6	661	69.1	52	5.4	18	1.9	957
	missing	5188	93.6	259	4.7	87	1.6	4	0.1	5	0.1	5543
	All	5188	46.9	1391	12.6	3923	35.5	382	3.5	178	1.6	11,062

### Asthma checker results

Self-reported outcomes from the monthly asthma checker results were compared for all users who had used the app at least twice where at least four months had elapsed between their first use and a subsequent use (n = 1581, range of number of uses = 2–30, median = 4, interquartile range = 4–8). Their first use was compared with all subsequent uses four or more months later. The following metrics were compared: those scoring an RCP three question of 0 and those using their reliever inhaler twice or less per week. Other collected data such as courses of prednisolone and emergency department attendances were not analysed due to the short period of comparison. This subgroup was generally older, and from less deprived areas than the overall cohort of app registrants (Table 3).

**Table 3:** Demographics of cohort of all app registrants and the 4 month+ app checker responders.

Characteristic	Level	0 uses		1 use		2+ uses <4 months apart		2+ uses 4+ months apart		Total downloads	
		n	*%*	n	*%*	n	*%*	n	*%*	n	*%*
Age	18–29	959	18.5	275	19.8	533	18.4	190	12.0	1957	17.7
	30–39	1163	22.4	282	20.3	560	19.3	263	16.6	2268	20.5
	40–49	970	18.7	274	19.7	611	21.1	294	18.6	2149	19.4
	50–59	1048	20.2	274	19.7	580	20.0	359	22.7	2261	20.4
	60–69	681	13.1	192	13.8	427	14.7	304	19.2	1604	14.5
	70+	347	6.7	91	6.5	183	6.3	166	10.5	787	7.1
	missing	20	0.4	3	0.2	3	0.3	5	0.3	36	0.3
	All	5188	100	1391	100	2902	100	1581	100	11,062	100
Practice deprivation group	Most 30%	1346	25.9	327	23.5	762	26.3	343	21.7	2778	25.1
	Next 20%	955	18.4	261	18.8	519	17.9	249	15.8	1984	19.7
	Least 50%	2731	52.6	757	54.4	1534	52.9	960	60.7	5982	54.1
	missing	156	3.0	46	3.3	87	3.0	29	1.8	318	2.9
	All	5188	100	1391	100	2902	100	1581	100	11,062	100

**Fig. 2:**
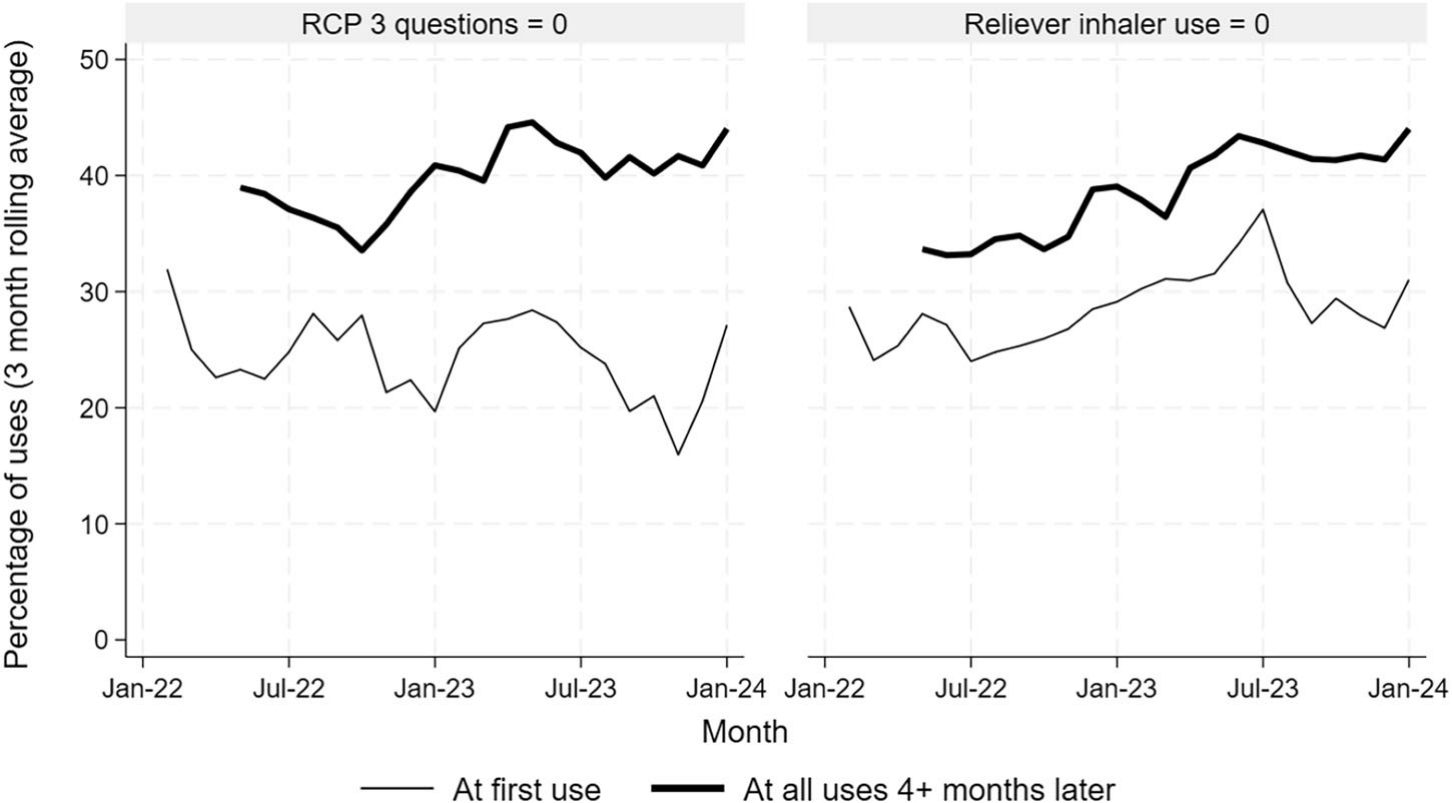
Outcomes at first app use compared to app uses four or more months later for RCP three question score and reliever usage.

The overall percent of this subgroup scoring zero for RCP3Q was 26.5% at their first app use and 40.7% for app uses four or more months later (difference 14.2%, 95% CI 11.3–17.0, *p* < 0.0001, Fig. 2). The overall percentages of this subgroup with zero uses of their reliever inhaler per week was 29.1% for at first app use and 39.2% for app uses four or more months later (difference 10.1%, 95% CI 7.2–13.0, *p* < 0.0001).

### Change in the RCP score at 12 months

We analysed paired data from the same asthma app users who recorded an RCP3Q in the corresponding month one year after their first RCP3Q was recorded. There were 133 app users of whom almost half were aged 60 or over, 11% were smokers and the least deprived 50% of practices were slightly overrepresented (Table 4). Overall there was a statistically significant improvement (mean difference −0.31, 95% confidence interval −0.52 to −0.09, paired t-test *p* = 0.0052) in RCP3Q scores at 12 month follow up. Seventeen percent of app users moved from potential sub-optimal or poor control to good control and another 17% improved their scores without reaching good control. These compare favorably with the 8% of app users who moved from good control to potential sub-optimal or poor control and the 9% whose scores worsened from a baseline of sub-optimal or poor control, whilst half recorded no change in their RCP score at 12 months. Age groups were collapsed into three categories. Improvement in RCP3Q scores at 12 months was statistically significant for category age 18–39 (mean difference −0.69, 95% confidence interval −1.20 to −0.18, paired t-test *p* = 0.0096) and an improvement was seen in all categories. Improvement in RCP3Q scores at 12 months was significant in non-smokers, and in smokers. All three deprivation groups showed an improvement in RCP3Q scores at 12 months. Statistical significance was reached for the most deprived 30% and the most deprived 30–50%, but not for the least deprived 50%.

**Table 4:** Changes in RCP3Q from baseline to 12 months.

Group	App users with both data points recorded		Change in RCP score at follow up			Paired t-test		
			Better than at baseline	Same as at baseline	Worse than at baseline	Mean difference (95% confidence interval)		p value
All app users	133		45 (33%)	66 (50%)	22 (17%)	−0.31	(−0.52 to −0.09)	0.0052
Ages 18–39	26	(20%)	14 (54%)	8 (31%)	4 (15%)	−0.69	(−1.20 to −0.18)	0.0096
Ages 40–59	49	(37%)	14 (29%)	27 (55%)	8 (16%)	−0.08	(−0.37 to 0.20)	0.57
Ages 60+	58	(44%)	17 (29%)	31 ((53%)	10 (17%)	−0.33	(−0.70 to 0.04)	0.082
Non-smokers	118	(89%)	38 (32%)	60 (51%)	20 (17%)	−0.26	(−0.49 to −0.03)	0.025
Smokers	15	(11%)	7 (47%)	6 (40%)	2 (13%)	−0.67	(−1.32 to −0.02)	0.045
Most deprived 30%	31	(23%)	8 (25%)	22 (71%)	1 (3%)	−0.42	(−0.77 to −0.07)	0.021
Most deprived 30–50%	18	(14%)	10 (56%)	7 (39%)	1 (6%)	−0.72	(−1.17 to −0.28)	0.0032
Least deprived 50%	82	(62%)	25 (30%)	37 (45%)	20 (24%)	−0.13	(−0.43 to 0.17)	0.37
missing	2	(2%)	2 (100%)					

## Discussion

In this paper, we present results from an initial evaluation of the national respiratory toolkit for Wales, concentrating on the impact of this intervention on self-reported markers of asthma control. The main findings are that for those who used the app there were improvements in good asthma control as evidenced by a self-reported RCP3Q score of zero as well as a reduction in reliever inhaler usage. When we compared the RCP3Q responses from the same users after one year, we found that there were greater improvements in those who moved from poor, or sub-optimal control to better control compared to those whose control worsened, but 50% showed no change. The improvements were significant in those from the most deprived 30% and 30–50%, but not in the least deprived 50%. This finding is of significant interest given the considerable concerns about health inequality and the uptake of digital solutions.

There has been considerable interest in developing digital solutions for people with asthma partly as a result of the findings of poor basic care for people with asthma from audits such as the National Respiratory Audit Program (NRAP) led by the RCP. In a rapidly expanding field, an early systematic review of the literature on the use of mobile applications to support self-management for people with asthma from 2017 showed improved asthma control but varied clinical effectiveness of different apps^17^. A more recent Cochrane review of digital interventions to improve adherence to maintenance medication in asthma showed that digital interventions were likely to improve adherence, asthma control and quality of life and may even reduce exacerbations^18^. The World Health Organisation advocates digital health technologies to advance population health^19^ and digital support tools have been shown to improve blood pressure control^20^. Given this evidence-based support for digital interventions, and with the very poor outcomes from the primary care RCP respiratory audit in Wales, it seemed logical to adopt a digital solution to support patients with their self-management. The major difficulty was in achieving adoption of this approach at a national level. We had previously implemented a dynamic guideline nationally in Wales during COVID rapidly gaining more than 13,000 healthcare professional (HCP) signups to the platform^10^. This same platform was used to deliver webinars about the asthma and other apps to a wide audience of HCP to prepare and inform them about this intervention.

One of the strengths of this study, is that it is a national approach. Every GP practice in Wales has people with asthma on the app with a median number of 21, but the best performing practice had 246 app registrants. Moreover, 88% of people with asthma are introduced to the app by their HCP, indicating that the apps are held to be of value within the health ecosystem. The results represent a ‘real world’ approach, outside the controlled confines of a research environment. It is not known what is the optimum frequency to use digital support tools, but we suspect that people with asthma use them when they need them, and that varies greatly from patient to patient.

There are many limitations to this study. Whist we have 12567 adults with asthma signed up to the app by September 2024, that represents only approximately 5% of all adults with asthma in Wales. Nevertheless, we note there is a continual linear increase in app signatories over time, without payment incentives or government directives to support adoption and we have engagement from all primary care practices. Indeed, it is well appreciated within implementation science literature that adoption of new interventions takes time, with one study suggesting that it takes on average 17 years for adoption of new research into routine clinical practice^21^. We note that 57.8% of patients who download the app go on to register, and of those who register, half do not use it at all. The reasons behind this are unclear, but we could postulate various explanations including that this group was better controlled and did not see a need for use, app ‘fatigue’, since people tend to have many apps but only use a small number regularly or perhaps that people agreed to download to show good intentions to their HCW, but with no intention of using it. Of interest is the finding that 52.2% of people who downloaded the NHS England app subsequently went on to register, despite the fact that this app provided crucial functions during COVID, such as demonstrating a user’s vaccination status^22^. The factors influencing behaviours around adoption of digital solutions such as apps will likely be a focus of future research among behavioural psychologists. We would hope that future developments in the app functions, including an ‘export’ function that allows relevant aspects of a person’s asthma control over the year such as requirements for prednisolone, hospital admissions and RCP score to be viewed by a HCP as part of an asthma annual review will increase uptake and engagement with the app. Another major limitation is that the results that we have described are patient responses and are not independently validated. However, what we have collected are patient recorded outcome measures (PROMS). PROMS have a long-established role in shaping health services to be responsive to patient feedback^23–25^, have been routinely embedded across a range of specialities, and lie at the heart of understanding value-based outcomes^26^. Although the PROMS that we have recorded are generated directly by the patients, rather than through questionnaires administered in healthcare settings, this may be a more appropriate approach for chronic conditions with fluctuating impacts like asthma.

Another limitation is that we only have responses on the 1591 users that had completed the asthma checker requests for four months or more, representing 12.7% of all app registrants. This likely reflects to some degree differences in asthma severity and control as well as variable engagement with the digital solution. In addition, we cannot exclude the fact that regression towards the mean may be a factor in the observed differences over four months or more of app usage, since those with more troublesome asthma would be more likely to engage with the app. We noted that the demographics of the asthma checker responders showed that those that engaged with the app were slightly older, and more came from less deprived areas than those of the asthma app registrants as a whole. However, registrants from more deprived areas seemed to benefit more, in terms of greater improvements in RCP3Q scores. Lastly, whilst we have collected and analysed data on some of the asthma checker responses, we have not included important metrics such as self-reported prednisolone courses, or emergency department attendances for asthma. We report here on an initial evaluation, and believe that this is too soon to evaluate the latter data.

In this study, we have implemented a digital solution for people with asthma in Wales without mandate or incentive. We note improvements as indicated by PROMS across a number of important metrics of asthma care, including the RCP3Q responses and reductions in reliever use for those that use the app. We noted greater engagement with the app for those from less deprived areas, but a greater improvement for those from more deprived areas, offering hope for the potential of digital solutions to reduce health inequalities^27^. These finding support our contention that helping patients to better manage their condition improves health outcomes.

## Data Availability

Data is provided within the manuscript.
